# Macrophage/microglia-producing transient increase of platelet-activating factor is involved in neuropathic pain

**DOI:** 10.1016/j.isci.2024.109466

**Published:** 2024-04-01

**Authors:** Shota Yamamoto, Tomomi Hashidate-Yoshida, Yuki Yoshinari, Takao Shimizu, Hideo Shindou

**Affiliations:** 1 Department of Lipid Life Science, National Center for Global Health and Medicine, Shinjuku-ku, Tokyo 162-8655, Japan; 2 Division of Molecular Neuroimmunology, Medical Institute of Bioregulation, Kyushu University, Fukuoka 812-8582, Japan; 3 Graduate School of Life Sciences, Showa Women’s University, Setagaya-ku, Tokyo, Japan; 4 Department of Lipid Signaling, National Center for Global Health and Medicine, Shinjuku-ku, Tokyo 162-8655, Japan; 5 Institute of Microbial Chemistry, Setagaya-ku, Tokyo, Japan; 6 Department of Medical Lipid Science, Graduate School of Medicine, The University of Tokyo, Bunkyo-ku, Tokyo, Japan

**Keywords:** Neuroscience, Immunology, Cell biology

## Abstract

Peripheral nerve injury (PNI) induces debilitating neuropathic pain symptoms, such as tactile allodynia. Accumulating evidence suggests that the expression levels of various transcripts and proteins are drastically changed after PNI. Recent lipidome analysis demonstrates increased levels of diverse lipids in chronic pain conditions. We show that PNI transiently increases platelet-activating factor (PAF) levels, a potent inflammatory phospholipid mediator, in the dorsal root ganglia (DRG) and spinal cord. We revealed that macrophage and microglia-specific PAF-producing enzyme LPLAT9/LPCAT2 knockout mice (*Cx3cr1*^CreERT2^;*Lpcat2*^flox/flox^) failed to develop mechanical allodynia and to increase PAF levels in the DRG and spinal cord after PNI. Moreover, we observed the suppression of PNI-induced PAF increase in the spinal cord of PAF receptor knockout mice, indicating a self-amplification loop of PAF production. In conclusion, macrophages and microglia enhance PAF production, contributing to PNI-induced neuropathic pain. Additionally, PAF-PAF receptor signaling is a potential target of neuropathic pain control.

## Highlights

• PAF levels are transiently increased in the DRG and spinal cord after nerve injury
• Macrophage/microglia-producing PAF contributes to PNI-induced neuropathic pain
• PAF production in the spinal cord is regulated by a self-amplification loop

## Graphical abstract

**Figure:**
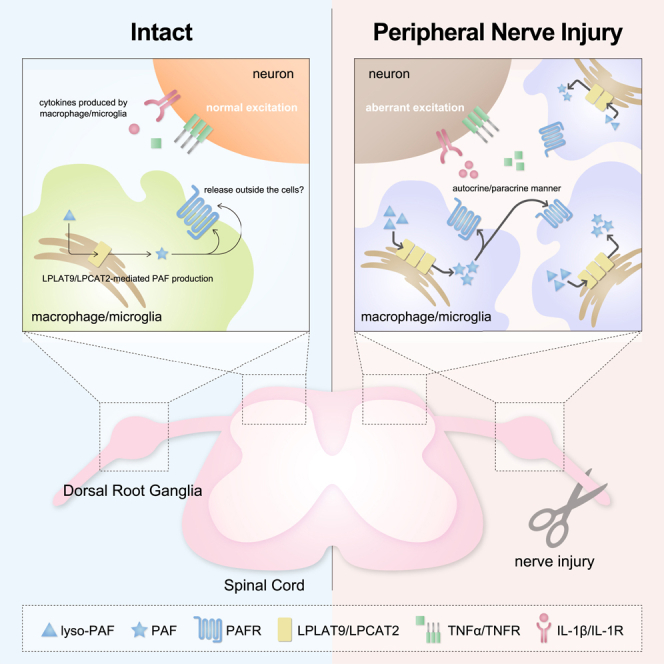

## Introduction

Neuropathic pain is characterized by debilitating chronic pain symptoms such as spontaneous pain, hyperalgesia, and allodynia and is often caused by damage to the nervous system resulting from cancer, chemotherapy, viral infection, autoimmune disease, and trauma.^1^ Despite the estimated 7%–10% prevalence of pathological pain syndromes among the human population,^2^ there is currently no effective treatment. To properly transmit sensory information to the brain, it requires cooperation between neurons and non-neuronal cells, including immune cells and glial cells, in the dorsal root ganglion (DRG) and spinal dorsal horn (SDH). However, this coordination is disrupted during pathological conditions, which leads to the development of allodynia and hyperalgesia.^3^^,^^4^

Accumulating evidence suggests that various chemokines and cytokines produced by both neurons and non-neuronal cells are upregulated after peripheral nerve injury (PNI), which results in neuroinflammation and the development of neuropathic pain.^4^^,^^5^^,^^6^ In addition, PNI increases a variety of lipid mediators in the DRG and spinal cord.^7^^,^^8^^,^^9^^,^^10^ Reportedly, various phospholipase A_2_ (PLA_2_) family enzymes, upstream enzymes in the production of lipid mediators, are upregulated and activated after PNI.^11^^,^^12^^,^^13^ This leads to an increase not only in fatty acid-derived metabolites (e.g., prostaglandins) but also bioactive mediators with phospholipid form such as lysophosphatidic acid (LPA) and platelet-activating factor (PAF) that are derived from phospholipids.^7^^,^^8^^,^^13^ Among these phospholipid mediators, the involvement of LPA in the pathology of neuropathic pain has been well studied.^10^^,^^14^^,^^15^ While further studies are needed to fully understand the biological roles of PAF in neuropathic pain. PAF is a potent inflammatory and immunological mediator generated in response to extracellular stimuli.^16^^,^^17^ Through Toll-like receptor 4, lipopolysaccharide upregulates PAF biosynthetic activity.^18^^,^^19^^,^^20^ The activity is also rapidly enhanced by ATP stimulation, which is one of main inducers of neuropathic pain.^20^^,^^21^^,^^22^ Reportedly, PNI-induced mechanical allodynia was attenuated in PAFR (PAF receptor)-deficient mice^23^ and biosynthetic enzyme LPCAT2 (lysophosphatidylcholine acyltransferase 2, also known as LPLAT9^19^^,^^24^)-deficient mice.^25^ However, many questions remain elusive regarding the spatiotemporal regulation of PAF in PNI pathological conditions, such as when and where PAF is produced and which cell types are responsible. To establish a PAF-signaling blocking strategy to control neuropathic pain, it is essential to determine specific time period and tissues to target.

In this study, we investigated the spatiotemporal changes in PAF levels after PNI. Furthermore, we also examined which cell types produce PAF in response to PNI in the DRG and spinal cord and how PAF contributes to the development and maintenance of neuropathic pain.

## Results

### PNI transiently increases PAF levels in both the DRG and spinal cord

**Figure 1:**
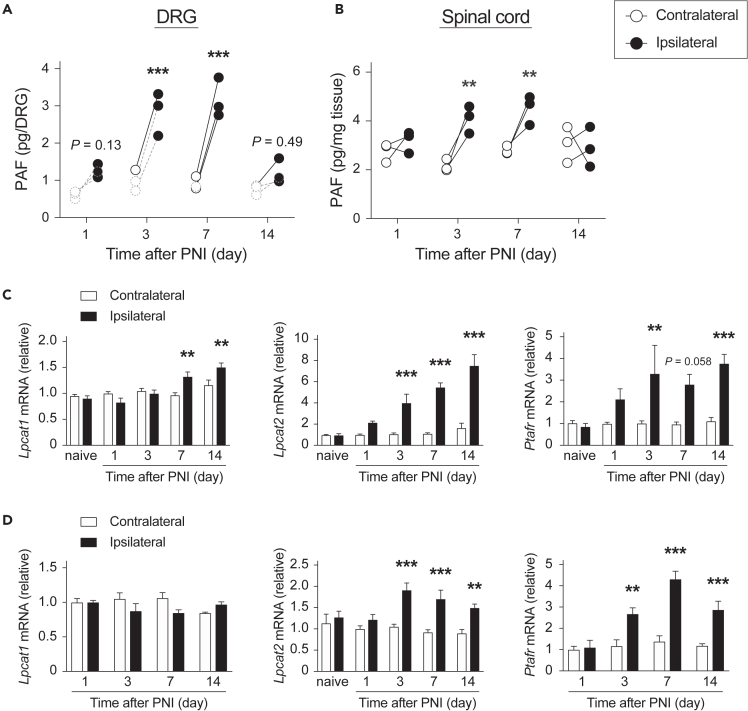
PNI increases PAF levels and expressions of PAF-related genes in the DRG and spinal cord (A and B) Quantification of platelet-activating factor (PAF) levels after PNI (A) in the DRG and (B) spinal cord (n = 3). (C and D) Expression levels of PAF-related genes (C) in the DRG and (D) spinal dorsal horn (n = 4–8). Dotted circle represents the value under the standard curve. **∗∗**p < 0.01, **∗∗∗**p < 0.001 vs. the contralateral side. Data are represented as mean ± SEM. *Lpcat1*, *Lpcat2*, and *Ptafr* mean gene names of LPLAT8, LPLAT9, and PAFR, respectively. See also Figure S1.

Firstly, we analyzed temporal changes in PAF levels after PNI in the DRG and spinal cord. We demonstrated that PNI transiently increased PAF levels from day 3 to day 7 in both tissues (Figures 1A and 1B) and that lyso-PAF, the precursor and metabolite of PAF, was similarly increased (Figures S1A and S1B). Moreover, we examined mRNA expression levels of PAF-related genes: *Lpcat1* (LPLAT8) and *Lpcat2* (LPLAT9) (biosynthetic enzymes), and *Ptafr* (PAFR) (receptor). We demonstrated that the expression levels of *Lpcat2* and *Ptafr* were significantly increased from 3 days after PNI in the DRG (Figure 1C) and SDH (Figure 1D). In addition, *Lpcat1* was slightly increased on day 7 and day 14 in the DRG (Figure 1C). These results suggest that PNI enhances PAF-PAFR signaling in both the DRG and spinal cord, and this effect is observed within a specific time frame (∼ day 7) after PNI.

### PAF-PAFR signaling is required for the maintenance and development of PNI-induced mechanical allodynia

**Figure 2:**
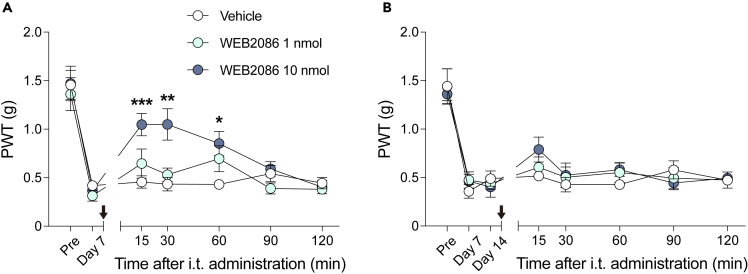
Difference in the effect of intrathecal WEB2086 injection against neuropathic mechanical allodynia (A and B) Paw withdrawal threshold (PWT) to mechanical stimuli is assessed by the von Frey test and calculated by the up-down method. WEB2086 (a PAF receptor antagonist, 1–10 nmol) was administrated intrathecally (A) on day 7, (B) day 14 after nerve injury (n = 5–7). Arrows indicate the timing of administration. **∗**p < 0.05, **∗∗**p < 0.01, **∗∗∗**p < 0.001 vs. vehicle. Data are represented as mean ± SEM. See also Figure S2.

In our PNI model used in the present study, it has demonstrated that mechanical allodynia developed from day 3 and was sustained until at least day 14.^7^^,^^26^ We examined the necessity of PAF-PAFR signaling in the maintenance phase of mechanical allodynia by using a PAFR antagonist (WEB2086). On day 7, intrathecal injection of WEB2086 (1–10 nmol) attenuated PNI-induced mechanical allodynia (Figure 2A). In addition, repeated treatment of WEB2086 on day 7 and 8 was also effective (Figure S2). Contrastingly, a WEB2086 treatment did not affect mechanical allodynia on day 14 (Figure 2B), on which PAF levels in the DRG and spinal cord have already returned to the equivalent levels to the contralateral side. These pharmacological experiments indicate that PAF-PAFR signaling can be involved in the maintenance of PNI-induced mechanical allodynia only while PAF levels are elevated.

**Figure 3:**
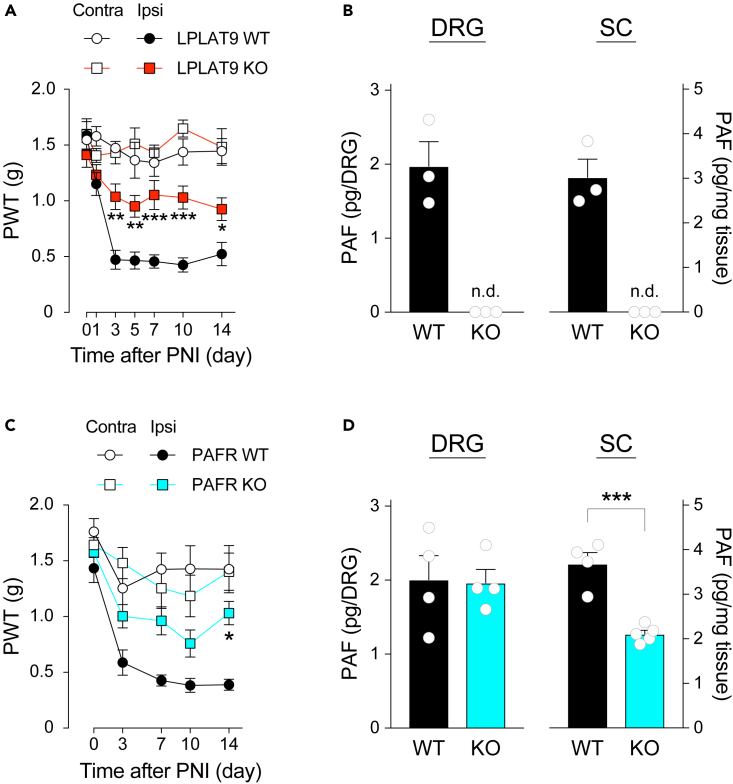
Deficiency of PAF signal-related genes prevents the development of mechanical allodynia and increase of PAF levels after PNI (A) PWT to mechanical stimuli of LPLAT9/LPCAT2 knockout (LPLAT9 KO) and littermate control (LPLAT9 WT) mice (n = 8–9). **∗**p < 0.05, **∗∗**p < 0.01, **∗∗∗**p < 0.001 vs. the ipsilateral side of LPLAT9 WT mice. (B) Quantification of PAF levels in the ipsilateral side of the DRG and spinal cord of LPLAT9 WT and KO mice 7 days after PNI (n = 3). **∗∗∗**p < 0.001 vs. LPLAT9 WT mice. N.D. indicates not detected and is regarded as zero for statistical analysis. (C) PWT to mechanical stimuli of PAFR knockout (PAFR KO) and littermate control (PAFR WT) mice (n = 7–9). **∗**p < 0.05 vs. the ipsilateral side of PAFR WT mice. (D) Quantification of PAF levels in the ipsilateral side of the DRG and spinal cord of PAFR WT and KO mice 7 days after PNI (n = 4–5). **∗∗∗**p < 0.001 vs. PAFR WT mice. Data are represented as mean ± SEM. See also Figures S3 and S4.

Subsequently, we also examined whether PAF-PAFR signaling is required for the developmental phase of PNI-induced mechanical allodynia by using LPLAT9-knockout (*Lpcat2*^−/−^) mice and PAFR-knockout (*Ptafr*^−/−^) mice. We observed that the development of mechanical allodynia was significantly suppressed in both of these mice (Figures 3A and 3C). However, LPLAT9 deficiency did not affect PNI-induced mechanical allodynia in female mice regardless of the fact that PNI similarly increased PAF levels in the DRG and spinal cord (Figure S3). Next, we measured PAF levels on day 7 after PNI in these mice. PAF in both the DRG and spinal cord of LPLAT9-knockout (LPLAT9 KO) mice were not detected as we previously reported the reduction of PAF levels in several LPLAT9 KO tissues (Figure 3B).^25^ In PAFR KO mice, although PNI-induced increase of PAF level in the DRG was comparable to that of wild-type mice, the increased PAF levels in the spinal cord were significantly attenuated (Figure 3D). Although *Lpcat1* mRNA in the DRG was increased after PNI, LPLAT8/LPCAT1 KO mice exhibited mechanical allodynia and increase of PAF levels in the DRG similar to wild-type mice (Figure S4). Intriguingly, in the spinal cord of LPLAT8 KO mice, PNI-induced increase of PAF level was enhanced (Figure S4). These results suggest that LPLAT9/LPCAT2-produced PAF-PAFR signaling is required not only to maintain but also to develop mechanical allodynia after PNI.

### LPLAT9/LPCAT2 is not required for the proliferation of macrophages and microglia

**Figure 4:**
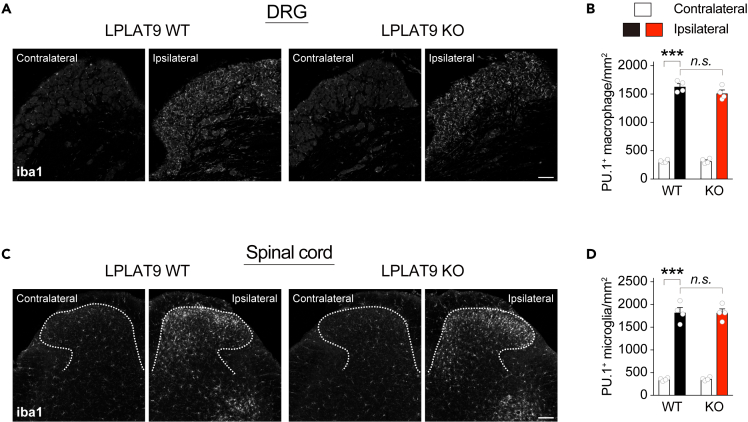
LPLAT9/LPCAT2 is not required for PNI-induced proliferation of DRG macrophages and spinal microglia (A) Immunofluorescence labeling of iba1 in the DRG of LPLAT9 WT and KO mice on day 7 after PNI (scale bar, 100 μm). (B) The density of PU.1^+^ macrophages in the neuronal cell body region of DRG 7 days after PNI (n = 4). **∗∗∗**p < 0.001 vs. the contralateral side of LPLAT9 WT mice. (C) Immunofluorescence labeling of iba1 in the spinal cord of LPLAT9 WT and KO mice on day 7 after PNI (scale bar, 100 μm). (D) The density of PU.1^+^ microglia in the surface dorsal horn region (the lateral side of PKCγ signals) 7 days after PNI (n = 4). **∗∗∗**p < 0.001 vs. the contralateral side of LPLAT9 WT mice. Data are represented as mean ± SEM. n.s., not significant.

In the DRG and SDH, drastic proliferation and morphological changes of macrophages and microglia occur in response to PNI, which are crucial for neuropathic pain.^3^^,^^4^ Therefore, we tested whether the deficiency of LPLAT9/LPCAT2 could impact PNI-induced cellular alterations of macrophages and microglia in the DRG and SDH, respectively, by using antibodies against a macrophage/microglial transcription factor PU.1 for counting individual nucleus. In the DRG, we found that LPLAT9 KO mice exhibited an equivalent increased cell number of macrophages compared with wild-type mice after PNI (Figures 4A and 4B). Moreover, there were no apparent differences between wild-type and LPLAT9 KO mice in PNI-induced microgliosis in the SDH (Figures 4C and 4D). These results indicate that LPLAT9/LPCAT2 is not required for the proliferation of macrophages and microglia after PNI.

### LPLAT9/LPCAT2 is abundantly expressed in macrophages and microglia

**Figure 5:**
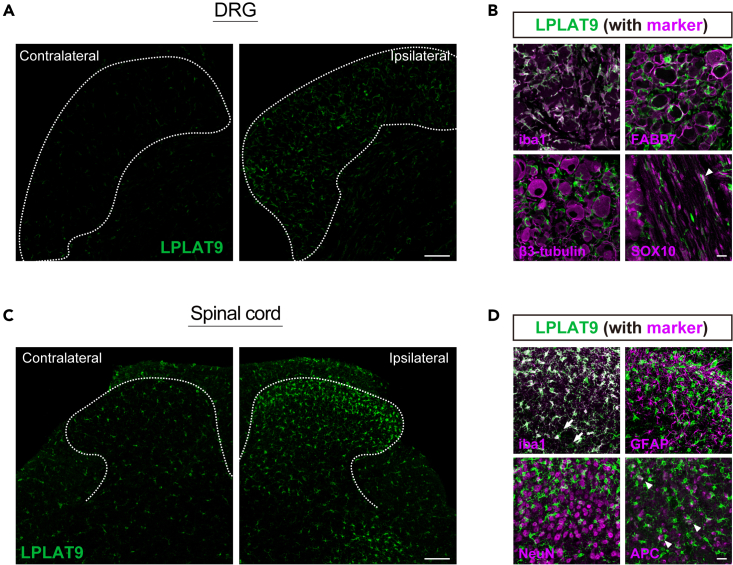
Immunohistochemistry for identification of cell types expressing LPLAT9/LPCAT2 in the DRG and spinal cord after PNI (A) Immunofluorescence labeling of LPLAT9 in the DRG on day 7 after PNI (scale bar, 100 μm). (B) Double-immunofluorescence labeling of cell type markers with LPLAT9 in the ipsilateral DRG 7 days after PNI (scale bar, 20 μm). Iba1 for macrophage, fatty acid-binding protein 7 (FABP7) for satellite glial cells, β3-tubulin for sensory neurons, and SOX10 for Schwann cells are used as cell type markers (arrowheads: SOX10^+^LPLAT9^+^ cells). (C) Immunofluorescence labeling of LPLAT9 in the spinal cord on day 7 after PNI (scale bar, 100 μm). (D) Double-immunofluorescence labeling of cell type markers with LPLAT9 in the ipsilateral spinal dorsal horn 7 days after PNI (scale bar, 20 μm). Iba1 for microglia, GFAP for astrocytes, NeuN for neurons), and APC for oligodendrocytes are used as cell type markers (arrowheads: APC^+^LPLAT9^+^ cells, arrows: iba1^–^LPLAT9^+^ cells). See also Figures S5 and S6.

We performed immunohistochemical staining by using an LPLAT9/LPCAT2-specific antibody, which was validated on the tissues from LPLAT9 KO mice (Figure S5), to identify the cell types that can produce PAF in response to PNI. We observed that PNI significantly increased LPLAT9 immunoreactivity in both the DRG and SDH on day 7 (Figures 5A and 5C), but not after sham treatment (Figure S6). In the DRG, coimmunostaining revealed that most of the LPLAT9^+^ cells co-expressed iba1 (a marker of macrophage) (Figure 5B). LPLAT9-expressing cells were partially overlapped with FABP7 (satellite glial cell) or SOX10 (Schwann cell), but not with β3-tubulin (sensory neuron) (Figure 5B). Moreover, we found that most of LPLAT9^+^ cells co-expressed iba1 (microglia) in the spinal cord (Figure 5D). A fraction of LPLAT9^+^ cells were overlapped with APC (oligodendrocyte), but not GFAP (astrocyte) or NeuN (neuron) (Figure 5D). These results indicate that PAF-producible LPLAT9-expressing cells are non-neuronal cells, particularly macrophages and microglia in the DRG and spinal cord, respectively. Furthermore, the increased expression levels of LPLAT9/LPCAT2 after PNI were due to the proliferation of macrophages and microglia.

### Macrophage/microglia-producing PAF is necessary to develop mechanical allodynia after PNI

**Figure 6:**
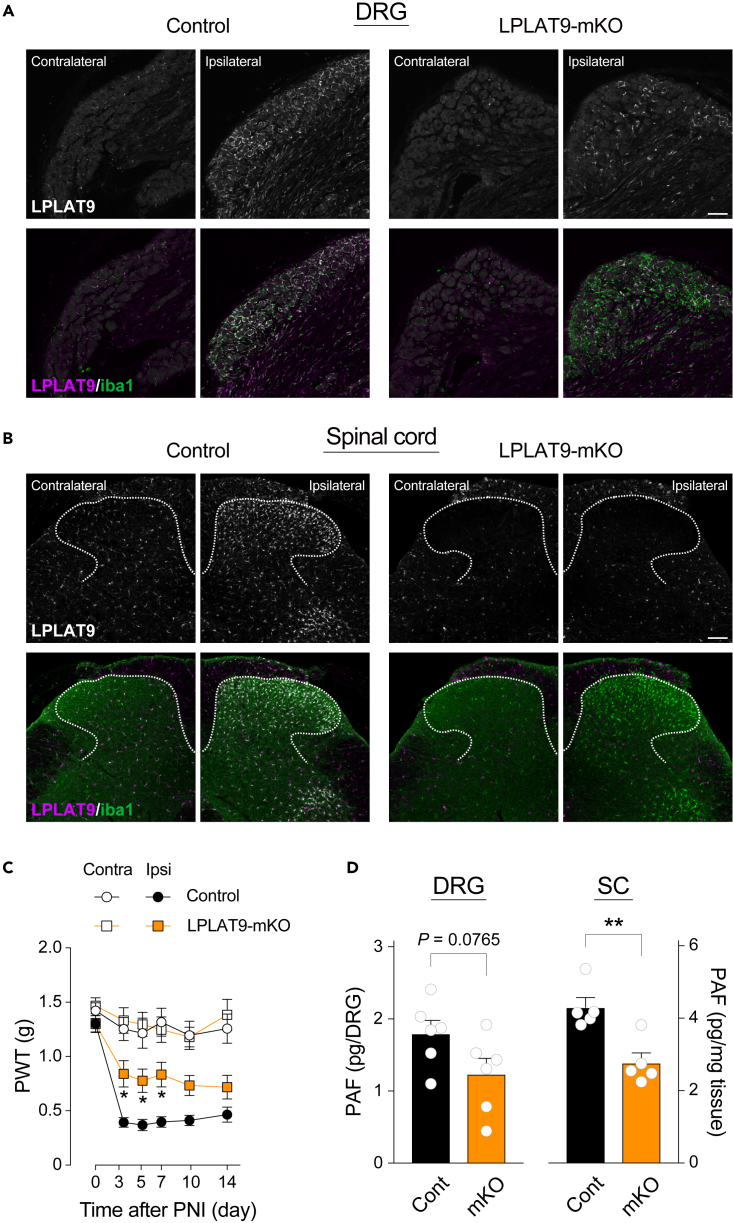
*Cx3cr1*^CreERT2^;*Lpcat2*^flox/flox^ mice fail to develop mechanical allodynia and increase PAF levels after PNI (A and B) Immunohistochemical validations of tamoxifen (TAM)-induced macrophage/microglia-specific LPLAT9/LPCAT2 conditional deletion (LPLAT9-mKO, *Cx3cr1*^CreERT2^;*Lpcat2*^flox/flox^) (A) in the DRG and (B) spinal cord of LPLAT9-mKO and littermate control (*Lpcat2*^flox/flox^) mice after PNI (scale bar, 100 μm). Upper panel: representative images of LPLAT9 staining. Lower panel: representative images of LPLAT9 (magenta) and iba1 (green). (C) PWT to mechanical stimuli of LPLAT9-mKO and littermate control (*Lpcat2*^flox/flox^) mice (n = 13–14). **∗**p < 0.05 vs. the ipsilateral side of control mice. (D) Quantification of PAF levels in the ipsilateral side of the DRG and spinal cord of control (Cont) and LPLAT9-mKO (mKO) mice 7 days after PNI (n = 5–6). **∗∗**p < 0.01 vs. control mice. Both genotypes were injected with TAM 4−5 weeks before PNI. Data are represented as mean ± SEM.

Finally, we established macrophage/microglia-specific LPLAT9/LPCAT2 knockout mice (LPLAT9-mKO, *Cx3cr1*^CreERT2^;*Lpcat2*^flox/flox^) to examine whether these cell types are responsible for the increased PAF levels and the development of mechanical allodynia after PNI. We subjected PNI to LPLAT9-mKO mice 4–5 weeks after TAM treatment because CX3CR1^+^-circulating monocytic cells are replaced by cells newly generated from bone-marrow-derived CX3CR1^–^ progenitors within this duration.^27^ On day 7 after PNI, we confirmed that a large population of iba1^+^ macrophages did not show immunoreactivity with anti-LPLAT9 antibodies in both the contralateral and ipsilateral sides of DRG (Figure 6A). In addition, almost all iba1^+^ microglia were recombined after TAM treatment in the gray matter of the SDH (Figure 6B). Behavioral analysis revealed that the development of PNI-induced mechanical allodynia was significantly attenuated in LPLAT9-mKO mice (Figure 6C). Consistently, the increased PAF levels after PNI were reduced in the DRG and spinal cord compared with those of control mice (*Lpcat2*^flox/flox^) (Figure 6D). In summary, these results indicate that LPLAT9/LPCAT2 expressed in macrophage/microglia is crucial for upregulation of PAF levels in the DRG and spinal cord after PNI, and also for the development of PNI-induced mechanical allodynia.

## Discussion

Neuropathic pain is commonly known to be unresponsive to non-steroidal anti-inflammatory drugs, which inhibit the production of prostaglandins.^1^^,^^28^ However, accumulative evidence has demonstrated that phospholipid-derived lipid mediators, such as LPA and sphingosine-1-phosphate, are involved in the pathogenesis of neuropathic pain and that inhibition of these receptor signaling could alleviate pain symptoms in several rodent models.^9^^,^^10^^,^^29^ Although PAF is also one of the most classical phospholipid-form lipid mediators,^30^^,^^31^ the spatiotemporal quantitative changes and cell types producing PAF in response to PNI remain unknown. In the present study, we revealed that PNI transiently increased PAF levels in both the DRG and spinal cord and that macrophage/microglia-produced PAF is responsible for the development of mechanical allodynia after PNI.

A previous lipidomic study demonstrated that tibial nerve injury in rats increased PAF levels in the spinal cord on day 21.^8^ In this study, we conducted a detailed temporal quantitative PAF analysis not only in the spinal cord but also in the DRG of mice with spinal nerve injury. Although there were some variations in the species (rat vs. mouse) and the PNI procedures (tibial nerve injury vs. spinal nerve injury) compared with the previous study, our findings revealed a transient increase in PAF levels in both tissues on days 3–7 after PNI, and then it returned to the control levels by day 14 despite the sustained existence of allodynia symptoms. Notably, the intrathecal injection of a PAFR antagonist attenuated mechanical allodynia only on day 7 but not on day 14 after PNI. These findings indicate that maintenance mechanisms of allodynia are shifted from PAF-PAFR signaling-dependent phase to the independent phase, such as neuronal or astrocytic alteration-dependent mechanisms, during days 7–14 after PNI. Furthermore, PAF injection causes not only mechanical allodynia but also thermal hypersensitivity,^23^^,^^32^ suggesting that PAF-PAFR signaling may be involved in thermal hyperalgesia after PNI. Moreover, we found sex difference in the phenotype of allodynia suppression in LPLAT9 KO mice. It is well known that sex differences in the role of microglia in neuropathic pain and spinal microglia may not be actively involved in the onset and maintenance of pain in females.^3^^,^^33^ Therefore, in female mice, PAF-PAFR signaling in microglia and macrophages is not required for the pathogenesis of neuropathic pain, but it is thought to be mediated by other mechanisms.

Our results showed that the alteration of PAF levels was not correlated with that of lyso-PAF levels or *Lpcat2* mRNA expressions. We previously demonstrated that in cultured macrophages, PAF biosynthetic activity of LPLAT9/LPCAT2 was drastically enhanced by phosphorylation at Ser-34 through Toll-like receptor 4 (TLR4)/p-38 mitogen-activated protein kinase (MAPK)-dependent pathway and/or PAF- and ATP-induced Ca^2+^/phospholipase Cβ/protein kinase Cα-dependent pathway.^18^^,^^19^^,^^21^ In the spinal cord, it has been reported that the expression levels of both TLR4 and phosphorylated p-38 MAPK in microglia are transiently upregulated after PNI.^34^^,^^35^ Moreover, accumulating evidence suggests that PNI-induced excessive calcium signaling in the DRG and SDH is critically contributed to the pathogenesis of neuropathic pain.^1^^,^^6^^,^^36^ Based on these observations, it is a plausible explanation of uncorrelated time course of PAF, lyso-PAF, and *Lpcat2* mRNA expressions that PAF levels after nerve injury may also be affected by the expression and phosphorylation levels of LPLAT9/LPCAT2. It is also possible that PNI-induced upregulation of *Lpcat2* mRNA expression occurs in other cell types expressing *Lpcat2* rather than macrophages/microglia. However, the upstream triggers for PNI-induced enhancement of PAF biosynthesis remain to be elucidated. PAF levels are also regulated by several PLA_2_s (lyso-PAF-producing enzyme) and PAF acetylhydrolases (PAF-AHs, PAF-hydrolyzing enzyme). Thus, further studies including evaluation of PLA_2_s/PAF-AHs activities are needed to understand PAF regulation under PNI conditions.

The local injection of PAF induces inflammation and pain in rodents^37^^,^^38^^,^^39^ and humans,^40^ which are PAFR-mediated effects.^23^^,^^32^ Additionally, intrathecal PAF injection increases the expressions of inflammatory cytokines such as tumor necrosis factor α (TNF-α) and interleukin-1β (IL-1β).^23^ Following PNI, it has been well demonstrated that a variety of inflammatory cytokines are released from macrophages and microglia.^4^^,^^6^ Furthermore, although there were no obvious differences in the PNI-induced increase of these cell numbers between wild-type and LPLAT9 KO mice in this study, LPLAT9 or PAFR deficiency possibly affects cellular states of macrophages/microglia. Actually, we previously revealed that the increase of TNF-α and IL-1β mRNA expressions after PNI was significantly suppressed in *Ptafr*^−/−^ mice.^23^ Hence, PAF-PAFR signaling-mediated increase of proinflammatory cytokines may result in neuronal hyperexcitability and subsequently contributes to the development of neuropathic pain.

Using cell culture experiments, we previously reported that there is a positive feedback loop in the production of PAF in response to PAFR stimulation.^25^ According to some studies using single-cell RNA sequencing (scRNA-seq) or *in situ* hybridization analysis, *Ptafr* mRNA specifically expresses on microglia in the spinal cord, which is a similar population expressing LPLAT9/LPCAT2.^41^^,^^42^ Furthermore, in the present study, we found that PNI-induced PAF elevation was significantly attenuated in the spinal cord of PAFR-deficient mice. The evidence strongly supports the *in vivo* existence of a positive feedback loop in PAF production. These mean that PAF produced by LPLAT9/LPCAT2-expressing microglia acts on PAFR in an autocrine/paracrine manner and is followed by new PAF production through LPLAT9 activation/induction, although it remains unknown whether PAF is released extracellularly. This self-amplification loop may regulate the PAF levels, at least in the spinal cord of mice, and contribute to neuropathic pain, potentially aligning with the proposed PAF-pain loop.^25^ Given that the PAF level in the DRG is not affected by the deficiency of PAFR, there should be distinct regulatory mechanisms of PAF levels in the DRG and spinal cord, although further analysis is needed.

In conclusion, we demonstrated the temporal profiles of PAF levels following PNI and the necessity of macrophage/microglia-producing PAF in the pathology of neuropathic pain. Addressing the aforementioned questions and developing novel analgesics targeting PAF-PAFR signaling blockade will be crucial for advancing pain management strategies.

### Limitations of the study

Finally, our findings raise several new questions that need further investigation. It remains unclear whether PNI-induced neuropathic pain requires the PAF increase in the DRG or spinal cord, or both. Recently, transcriptional differences among DRG- or peripheral nerve-resident macrophages and microglia have been shown using scRNA-seq,^43^^,^^44^ indicating that the microglia-associated gene *Sall1* is absent or less expressed in DRG- or peripheral nerve-resident macrophages. In addition, microglia do not express CD206 during adulthood (encoded by *Mrc1*), whereas the majority of DRG macrophages are positive for CD206.^42^^,^^45^^,^^46^^,^^47^ Although further investigations are needed, *Sall1*^CreERT2^^48^ or *Mrc1*^CreERT2^^47^^,^^49^ mice lines might have possibilities to manipulate selectively between DRG-resident macrophage and spinal cord microglia, which are enabled to elucidate the aforementioned question. Furthermore, the role of LPLAT9 expressed in non-neuronal cells other than macrophages/microglia also deserves to be studied because these cell types are known to be involved in various physiology and neurological diseases. Additional question is raised from the present finding that PAF-PAFR signaling is required only during certain periods of neuropathic pain. Therefore, how can we know when to block PAF-PAFR signaling for pain control? Future studies should aim to identify biomarkers that reflect the specific phase of the pathology.

## STAR★Methods

### Key resources table

**Table:** 

REAGENT or RESOURCE	SOURCE	IDENTIFIER
**Antibodies**		

rabbit anti-PU.1	Cell Signaling Technology	Cat# 2258, RRID:AB_2186909
rabbit anti-iba1	Wako	Cat# 019-19741, RRID:AB_839504
guinea pig anti-iba1	Synaptic Systems	Cat# 234 004, RRID:AB_2493179
guinea pig anti-iba1	Synaptic Systems	Cat# 234 308, RRID:AB_2924932
goat anti-PKCγ	Frontier Institute	Cat# PKCg-Go-Af840, RRID:AB_2571825
rabbit anti-LPLAT9/LPCAT2	Atlas Antibodies	Cat# HPA007891, RRID:AB_1845223
rabbit anti-LPLAT9/LPCAT2	Invitrogen	Cat# PA5-52481, RRID:AB_2643435
mouse anti-β3 tubulin	BioLegend	Cat# 801202, RRID:AB_10063408
goat anti-FABP7	R&D systems	Cat# AF3166, RRID:AB_2100475
goat anti-SOX10	R&D systems	Cat# AF2864, RRID:AB_442208
mouse anti-NeuN	Abcam	Cat# ab104224, RRID:AB_10711040
Rat anti-GFAP	Invitrogen	Cat# 13-0300, RRID:AB_2532994
mouse anti-APC	Millipore	Cat# OP80-100UG, RRID:AB_2057371

**Chemicals, peptides, and recombinant proteins**		

tamoxifen	Sigma	Cat# T5648
C16-PAF-d4	Cayman	Cat# 360900
lyso-PAF-d4	Cayman	Cat# 360906
WEB2086	Tocris Bioscience	Cat# 2339

**Experimental models: Organisms/strains**		

C57BL/6 mice	Clea Japan	N/A
LPLAT9/LPCAT2-deficient (Lpcat2–/–) mice	Shindou et al.^25^	N/A
LPLAT8/LPCAT1-deficient (Lpcat1–/–) mice	Harayama et al.^50^	N/A
PAFR-deficient (Ptafr–/–) mice	Ishii et al.^51^	N/A
B6.129P2(Cg)-*Cx3cr1*^*tm2.1(cre/ERT2)Litt*^/WganJ	The Jackson Laboratory	RRID:IMSR_JAX:021160
Lpcat2-floxed mice	Shindou et al.^25^	N/A

**Software and algorithms**		

LabSolutions Insight	Shimadzu	Cat# 225-32701-91
GraphPad Prism 7	GraphPad	https://www.graphpad.com/

**Other**		

Oasis HLB column	Waters	Cat# 186000383
LCMS-8060	Shimadzu	Cat# 225-27800-41K
reverse-phase column (Kinetex C8)	Phenomenex	Cat# 00F-4497-AN

### Resource availability

#### Lead contact

Further information and requests for resources and reagents should be directed to Hideo Shindou and will be fulfilled by the lead contact, Hideo Shindou, hshindou@ri.ncgm.go.jp.

#### Materials availability

This study did not generate new unique reagents.

#### Data and code availability

• Accession numbers are listed in the key resources table.
• This paper does not report original code.
• Any additional information required to reanalyze the data reported in this paper is available from the lead contact upon request.

### Experimental model and study participant details

C57BL/6 mice were purchased from Clea Japan (Japan). LPLAT9/LPCAT2-deficient (*Lpcat2*^–/–^) mice,^25^ LPLAT8/LPCAT1-deficient (*Lpcat1*^–/–^) mice,^50^ PAFR-deficient (*Ptafr*^–/–^) mice,^51^ and their wild-type mice were used. To generate macrophage/microglia-specific knockout mice, *Cx3cr1*^CreERT2^ mice [B6.129P2(Cg)-*Cx3cr1*^*tm2.1(cre/ERT2)Litt*^/WganJ: The Jackson Laboratories, Strain number 021160] were crossed with *Lpcat2*-floxed mice.^25^ All mice used were aged 8–15 weeks at the start of each experiment, and were housed with lights on from 8:00 to 20:00 and were fed food and water *ad libitum*. Male mice were used in all experiments except as noted. All animal experiments were conducted according to the guidelines of the Animal Research Committee of the National Center for Global Health and Medicine, using protocols approved by the committee (approved number: 2023-A036).

### Method details

#### Tamoxifen treatment

For induction of Cre recombinase activity, 4–6-week-old *Cx3cr1*^CreERT2^ mice were injected subcutaneously with 2 mg tamoxifen (TAM) (Sigma) dissolved in corn oil (Wako), twice at approximately 48 hours intervals.^52^ To avoid the influence of circulating monocytic cells, we performed the PNI procedure 4–5 weeks after TAM administration.^27^

#### Peripheral nerve injury

As a neuropathic pain model, we used the spinal nerve injury model with some modifications.^7^ Briefly, mice were anesthetized with 2–3% isoflurane, and a small incision was made on the back. The L5 transverse process was removed to expose the L4 spinal nerve. The exposed L4 spinal nerve was carefully cut, and the wound and skin were sutured with 5-0 silk. Sham surgery was performed according to the above procedure without spinal nerve cut.

#### Sample preparation for PAF and lyso-PAF measurements

Mice were deeply anesthetized and perfused with ice-cold saline from the left ventricle. The L4 DRG and L3/4 spinal cord were quickly removed and frozen in liquid nitrogen. We made pooled samples of these tissues combined with 2–6 mice because we could not detect any signals indicating PAF in the liquid chromatography-mass spectrometry (LC-MS) measurement due to its very low amount. Frozen tissues were pulverized, and lipid components were extracted for 60 min at 4°C in methanol spiked with deuterium-labeled C16-PAF-d4 and lyso-PAF-d4 (Cayman) as the internal standard. After centrifugation at 15,000 × *g* for 10 min, the supernatants were collected. Methanol extracts were purified by a solid-phase method with the Oasis HLB column (Waters).

#### PAF and lyso-PAF measurement

Endogenous PAF and lyso-PAF were measured using a triple quadrupole mass spectrometer LCMS-8060 (Shimadzu, Japan) as previously described.^25^ In brief, a reverse-phase column (Kinetex C8, 2.1 × 150 mm, 2.6 μm, Phenomenex) was used for chromatographic separation with a binary mobile phase of the following compositions: 0.1% formic acid/water (mobile phase A) and acetonitrile (mobile phase B). The gradient of the mobile phase (%A/%B) was programmed as follows: 0 min (90/10), 5 min (75/25), 10 min (65/35), 20 min (25/75), 20.1–28 min (5/95), 28.1–30 min (90/10). The flow rate was 0.4 mL/min, and the column temperature was 40°C. The selected reaction-monitoring transitions were: *m/z* 568.4 → 59.1 (PAF), *m/z* 572.4 → 59.1 (PAF-d4), *m/z* 482.3 → 104.2 (lyso-PAF), and *m/z* 486.3 → 104.2 (lyso-PAF-d4). Raw data were analyzed using LabSolutions Insight (Shimadzu), and the signals were compared with those of standard curves for quantification. The calculated data are listed in Table S1.

#### Quantitative real-time PCR

Mice were deeply anesthetized and perfused transcardially with ice-cold saline, followed by an RNA stabilization solution (Ambion). The L4 DRG and SDH of the L3–4 segments were quickly removed and immersed in an RNA stabilization solution. Tissues were homogenized in QIAzol Lysis Reagent (QIAGEN), and total RNA was extracted using RNeasy Micro Kit (QIAGEN). We used the SuperScript III enzyme for reverse transcription. cDNA was then subjected to quantitative PCR using Fast SYBR Green Master Mix and the Step One Plus real-time PCR system (Applied Biosystems). The gene expressions were normalized with the value for *Gapdh*. The sequences of each primer pair are described below.

*Lpcat1*: 5ʹ-TCCCAGACCTTAGCCACCAT-3ʹ (forward), 5ʹ-ACAGGTTGGCCTCATCTATGCT-3ʹ (reverse).

*Lpcat2*: 5ʹ-CCCTGCCAATACAGAAGAGATCA-3ʹ (forward), 5ʹ-GCCGTCCTCATCAACATCAA-3ʹ (reverse).

*Ptafr*: 5ʹ-AGCAGAGTTGGGCTACCAGA-3ʹ (forward), 5ʹ-TGCGCATGCTGTAAAACTTC-3ʹ (reverse).

*Gapdh*: 5ʹ-TGACAATGAATACGGCTACAGCA-3ʹ (forward), 5ʹ-CTCCTGTTATTATGGGGGTCTGG-3ʹ (reverse).

#### Drug administration

For intrathecal (i.t.) injection, spinal cord puncture was made under isoflurane, with a 30 G needle attached to a 25-μL Hamilton syringe between the L5 and L6 of the spinal column to deliver reagent (5 μL) to the cerebrospinal fluid. As a PAFR antagonist, WEB2086 (1–10 nmol/mouse, Tocris Bioscience) was used.

#### Behavioral test

Mechanical sensitivity was assessed using the von Frey test.^7^ Briefly, each mouse was placed in a wire mesh cage and habituated for more than an hour before testing. Calibrated von Frey filaments (0.02–2.0 g) were applied to the mid-plantar skin of each hind paw. The 50% paw withdrawal threshold was determined using the up-down method.^53^ All behavioral assessment were not performed blind to experimental conditions.

#### Immunohistochemistry

Mice were deeply anesthetized and perfused transcardially with ice-cold saline, followed by 4% paraformaldehyde (PFA) (nacalai tesque). The L3/4 spinal cord and L4 DRG were removed and immersed in PFA for 3 hours at 4°C and then placed in 30% sucrose/phosphate-buffered saline (PBS) (nacalai tesque). Tissues were embedded in OCT compound (Sakura finetek) and frozen on liquid nitrogen, which was stored at –80°C until we used them. Tissue sections were made at 15 μm (DRG) or 30 μm (spinal cord) thickness. Sections were permeabilized and blocked in 3% normal goat or donkey serum/PBS containing 0.3% Triton X-100 (Wako) for 1–2 hours at room temperature. The primary antibodies were reacted at 4°C, and then, the corresponding secondary antibodies (Invitrogen or Jackson ImmunoResearch, 2 μg/mL) were used. The following primary antibodies have been used: rabbit anti-PU.1 (Cell Signaling Technology, 2258S, 1:1000), rabbit anti-iba1 (Wako, 019-19741, 1:2000), guinea pig anti-iba1 (Synaptic Systems, 234004 or 234308, 1:500), goat anti-PKCγ (Frontier Institute, PKCγ-Go-Af840, 1:500), rabbit anti-LPLAT9/LPCAT2 (Atlas Antibodies, HPA007891, 1:200−1:1000), rabbit anti-LPLAT9/LPCAT2 (Invitrogen, PA5-52481, 1:1000−1:2000), mouse anti-β3 tubulin (Biolegend, 801202, 1:2000), goat anti-FABP7 (R&D systems, AF3166, 1:1000), goat anti-SOX10 (R&D systems, AF2864, 1:1000), mouse anti-NeuN (Abcam, ab104224, 1:1000), Rat anti-GFAP (Invitrogen, 13-0300, 1:1000), mouse anti-APC (Millipore, OP80-100UGCN, 1:100). After washing step, the sections were mounted with VECTASHIELD (Vector Laboratories). Each section was observed using a confocal microscope LSM880 or LSM900 (Carl Zeiss) and 2−4 sections from the L4 DRG and spinal cord of each mouse, respectively, were randomly selected and analyzed with Fiji (https://fiji.sc). For quantification of the number of macrophages/microglia, PU.1^+^ nucleus in the region of interest (ROI), which were cell body area of DRG neurons and lamina I−II of the gray matter of SDH, were manually counted. ROI was determined from immunofluorescence of β3 tubulin (DRG) and PKCγ (inner lamina II of the SDH), respectively.

### Quantification and statistical analysis

Statistical analyses were performed using GraphPad Prism 7 software to determine differences among the groups. Data were analyzed using the Student’s t-test (Figures 3B, 3D, and 6D) or one-way ANOVA with *post hoc* Tukey’s test (Figures 4B and 4D) after determining the normality (Shapiro-Wilk test), or two-way ANOVA with *post hoc* Bonferroni test (Figures 1, 2, 3A,C, and 6C). A probability level of *P* < 0.05 was deemed statistically significant.

## Supplemental information

Document S1. Figures S1‒S6

Table S1. Raw datasets of PAF levels in the DRG and spinal cord after PNI, related to Figure 1

Table S2. Raw datasets of PAF levels in the DRG and spinal cord of LPLAT9 KO and PAFR KO mice after PNI, related to Figure 3

Table S3. Raw datasets of PAF levels in the DRG and spinal cord of macrophage/microglia-specific LPLAT9 KO mice after PNI, related to Figure 6

Table S4. Raw datasets of lyso-PAF levels in the DRG and spinal cord after PNI, related to Figure S1

Table S5. Raw datasets of PAF levels in the DRG and spinal cord of C57BL6 female mice after PNI, related to Figure S3

Table S6. Raw datasets of PAF levels in the DRG and spinal cord of LPLAT8 KO mice after PNI, related to Figure S4
