# Supplementary material for: Macrophage/microglia-producing transient increase of platelet-activating factor is involved in neuropathic pain
Source: iScience. 2024 Apr 1;27(4):109466. doi: 10.1016/j.isci.2024.109466 (PMC11074981; doi:10.1016/j.isci.2024.109466)
Supplement: Document S1. Figures S1‒S6 [file mmc1.pdf]

iScience, Volume *27*

Supplemental information

Macrophage/microglia-producing transient increase of platelet-activating factor is involved in neuropathic pain

Shota Yamamoto, Tomomi Hashidate-Yoshida, Yuki Yoshinari, Takao Shimizu, and Hideo Shindou

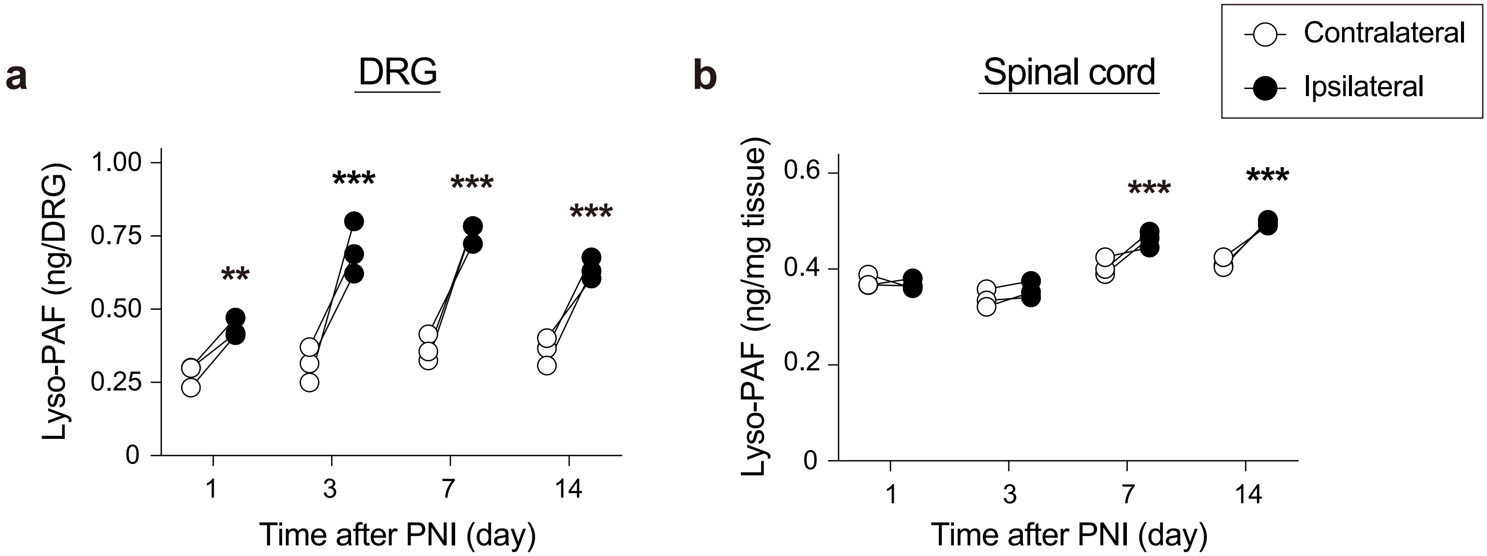

Supplementary Figure 1

PNI increases lyso-PAF levels in the DRG and spinal cord, but does not correlate with PAF levels, related to Figure 1. (a, b) Quantification of lyso-PAF levels after PNI (a) in the DRG and (b) spinal cord (n = 3). ***P* < 0.01, ****P* < 0.001 vs. the contralateral side. Data is represented as mean ± SEM.

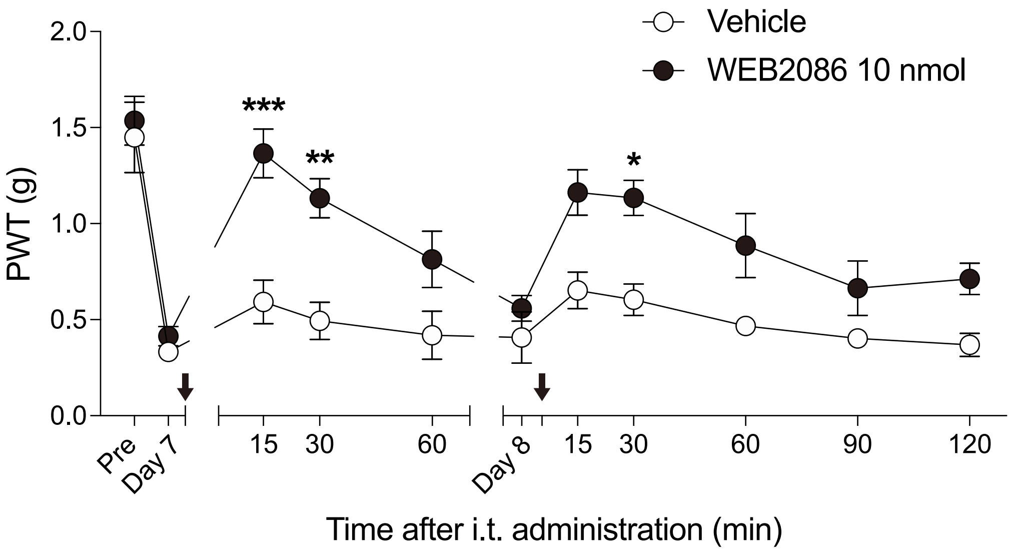

Supplementary Figure 2

Effect of repeated injection of WEB2086 against neuropathic mechanical allodynia, related to Figure 2. Paw withdrawal threshold (PWT) to mechanical stimuli is assessed by the von Frey test and calculated by the up-down method. WEB2086 (a PAF receptor antagonist) was administrated intrathecally on day 7 and 8 after nerve injury (n = 4−8). Arrows indicate the timing of administration. *P < 0.05, **P < 0.01, ***P < 0.001 vs. vehicle. Data is represented as mean ± SEM.

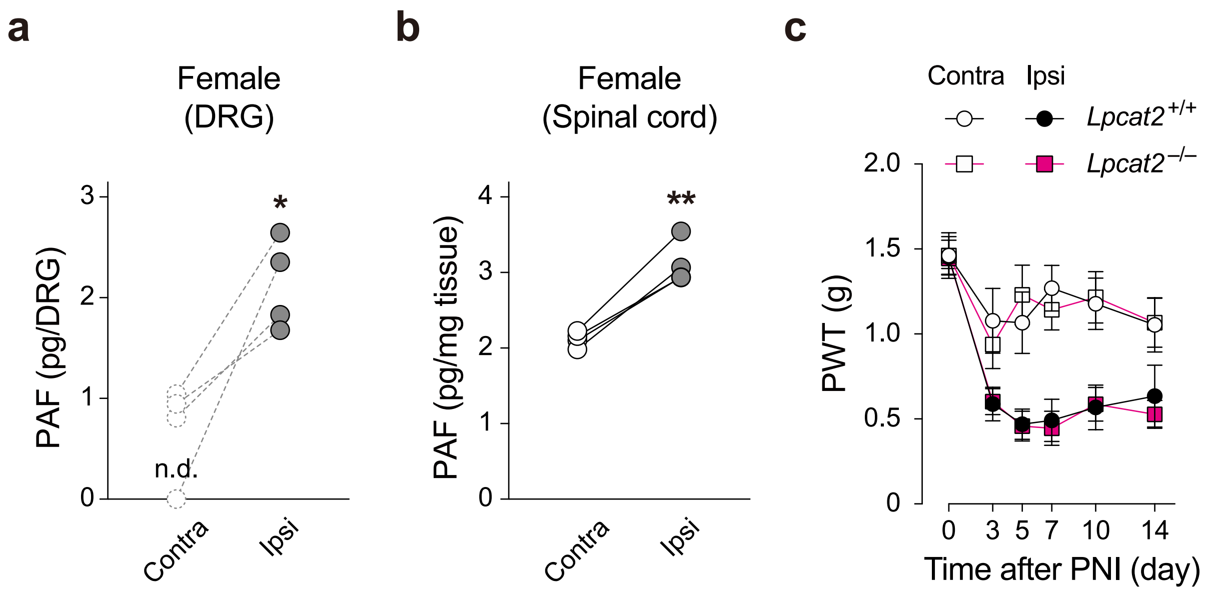

Supplementary Figure 3

PAF levels are increased after PNI in female mice but deficiency of LPLAT9/LPCAT2 does not affect the development of mechanical allodynia, related to Figure 3. (a) Quantification of PAF levels in the DRG and spinal cord of female C57BL6 mice 7 days after PNI (n = 4). Dotted circle represents the value under the standard curve. **P* < 0.05, ***P* < 0.01 vs. contralateral side. (b) PWT to mechanical stimuli of LPLAT9/LPCAT2 knockout (LPLAT9 KO) and littermate control (LPLAT9 WT) mice (n = 8−9). Data is represented as mean ± SEM.

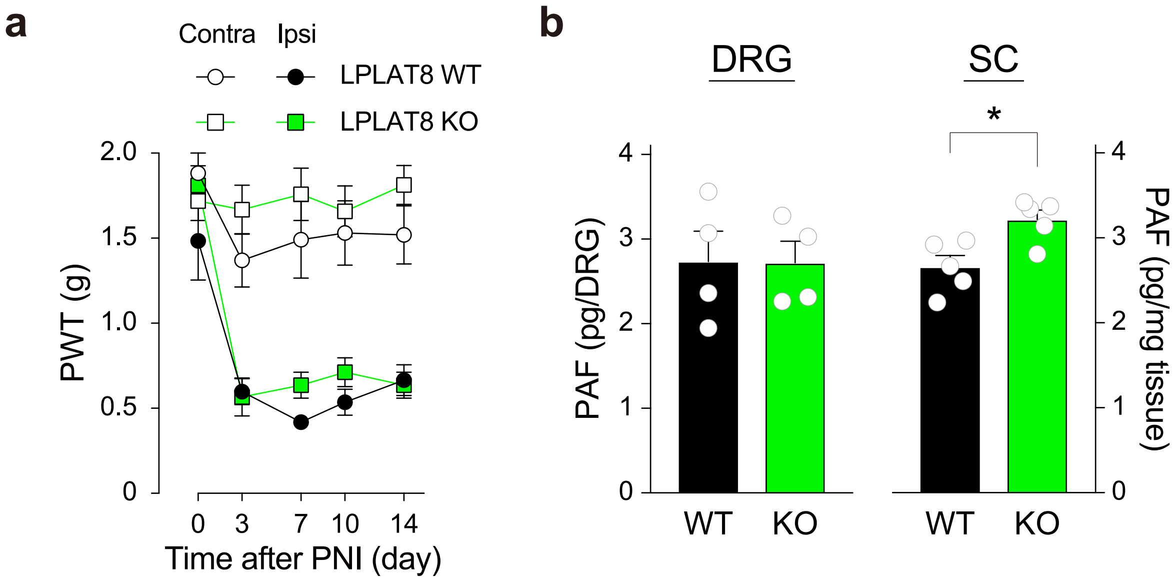

Supplementary Figure 4

Deficiency of LPLAT8/LPCAT1 does not affect the development of mechanical allodynia and increase of PAF levels after PNI, related to Figure 3. (a) PWT to mechanical stimuli of LPLAT8/LPCAT1 knockout (LPLAT8 KO) and littermate control (LPLAT8 WT) mice (n = 4−5). (b) Quantification of PAF levels in the ipsilateral side of the DRG and spinal cord of LPLAT8 WT and KO mice 7 days after PNI (n = 4−5). **P* < 0.05 vs. LPLAT8 WT mice. Data is represented as mean ± SEM.

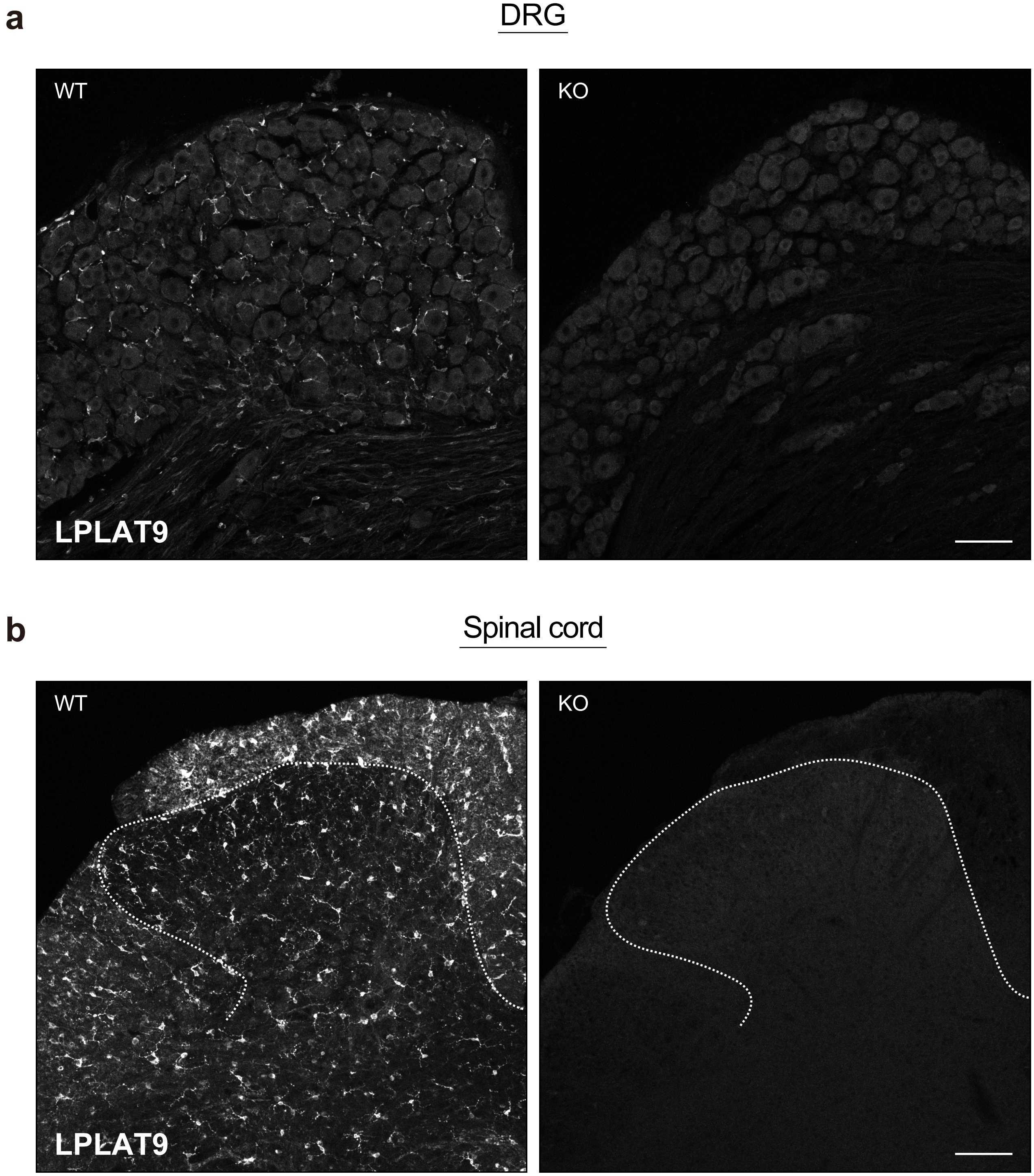

Supplementary Figure 5

Validations of LPLAT9/LPCAT2 antibodies on the DRG and spinal cord tissues, related to Figure 5. (a,b) Immunohistochemical validations of specificity of LPLAT9/LPCAT2 antibodies used in this study (a) in the DRG and (b) spinal cord (scale bar, 100 µm). Both male and female mice (WT and KO) were used in these experiments.

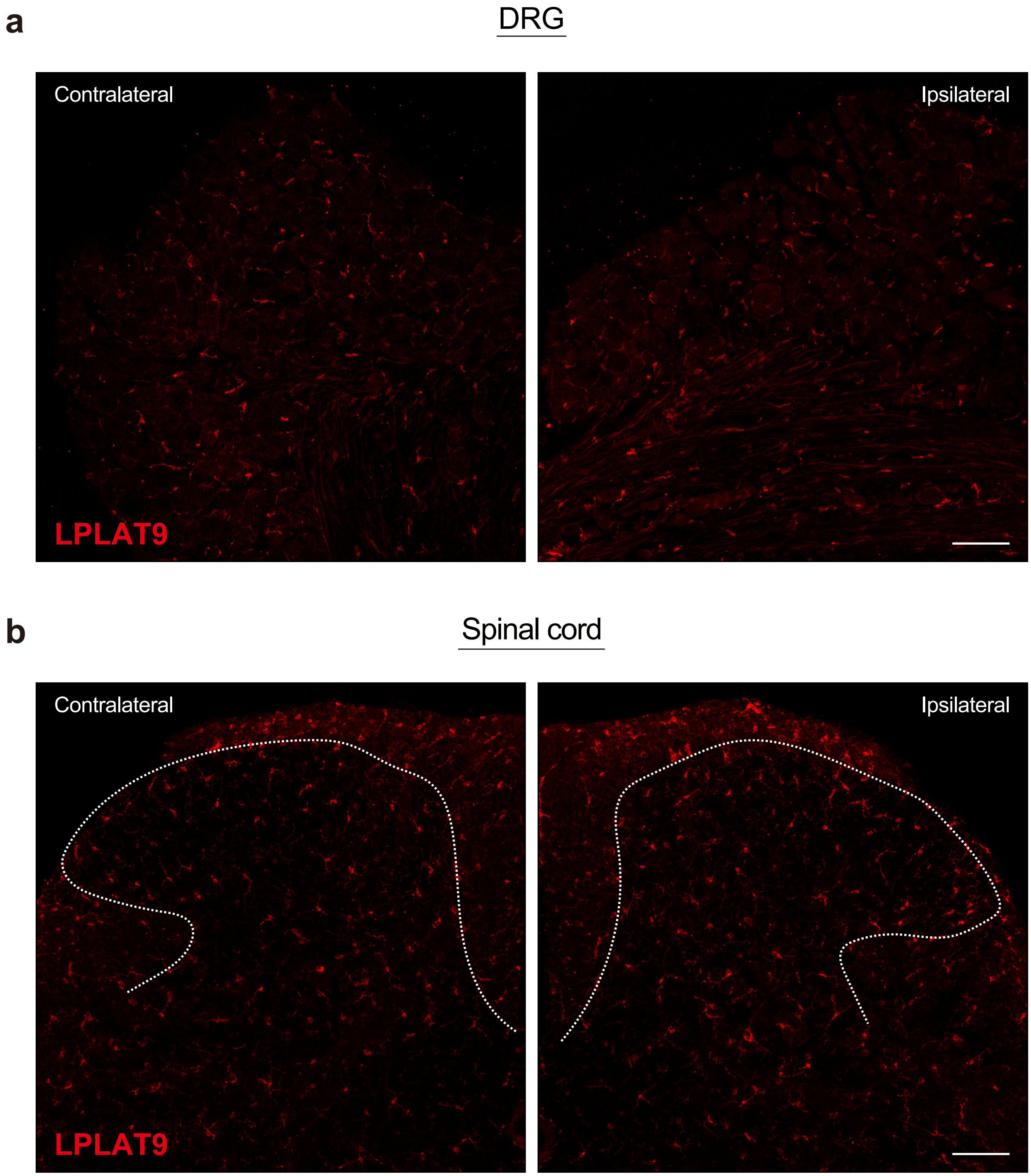

Supplementary Figure 6

PNI-sham operation does not affect the expression of LPLAT9/LPCAT2 in the DRG and spinal cord, related to Figure 5. (a,b) Immunohistochemical analysis about the LPLAT9/LPCAT2 expression levels (a) in the DRG and (b) spinal cord (scale bar, 100 μm).
